# Novel bioactive natural products from bacteria via bioprospecting, genome mining and metabolic engineering

**DOI:** 10.1111/1751-7915.13398

**Published:** 2019-03-04

**Authors:** Olga N. Sekurova, Olha Schneider, Sergey B. Zotchev

**Affiliations:** ^1^ Department of Pharmacognosy University of Vienna Althanstraße 14 1090 Vienna Austria

## Abstract

For over seven decades, bacteria served as a valuable source of bioactive natural products some of which were eventually developed into drugs to treat infections, cancer and immune system‐related diseases. Traditionally, novel compounds produced by bacteria were discovered via conventional bioprospecting based on isolation of potential producers and screening their extracts in a variety of bioassays. Over time, most of the natural products identifiable by this approach were discovered, and the pipeline for new drugs based on bacterially produced metabolites started to run dry. This mini‐review highlights recent developments in bacterial bioprospecting for novel compounds that are based on several out‐of‐the‐box approaches, including the following: (i) targeting bacterial species previously unknown to produce any bioactive natural products, (ii) exploring non‐traditional environmental niches and methods for isolation of bacteria and (iii) various types of ‘genome mining’ aimed at unravelling genetic potential of bacteria to produce secondary metabolites. All these approaches have already yielded a number of novel bioactive compounds and, if used wisely, will soon revitalize drug discovery pipeline based on bacterial natural products.

## Introduction

Natural products from microorganisms are structurally very diverse and represent a rich source for the discovery of new drugs to treat various human diseases, including infections and cancer. Over 50% of all pharmaceutical drugs currently on the market are directly derived from or inspired by natural products (Newman and Cragg, 2016). Bacteria are perhaps the most prolific microbial producers of bioactive natural products, which are represented by their secondary metabolites. The latter compounds are not required for the normal proliferation of the producing organisms, but appear to give some advantages in environmental adaptation and survival (Jenke‐Kodama *et al*., 2008; Strachan and Davies, 2016). Some studies suggest that secondary metabolites with antibiotic activity may be used by bacteria to fight off predators, to compete for nutritional sources (van der Meij *et al*., 2017) or even to defend their hosts (Samuels *et al*., 2013). Whatever the biological role(s) of secondary metabolites is in nature, they are important for the bacteria that synthesize them, since a significant amount of cellular resources, including building blocks from primary metabolism, cofactors and energy in the form of ATP and NADPH, are required for their biosynthesis. Apparently, the biosynthetic pathways for secondary metabolites evolved over millions of years to generate chemical structures that interact with particular biological targets, either in the producing organism itself or in those present in the surrounding environment. Consequently, it can be assumed that most, if not all, secondary metabolites have biological activities, although we may not yet have assays to detect these.

**Figure 1:**
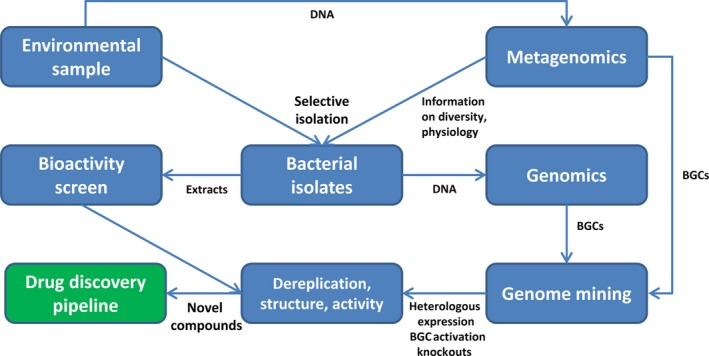
Overview of the workflow for the discovery of bioactive natural products from bacteria.

Considering the looming crisis of antibiotic resistance that spreads among bacterial pathogens and increasing incidence of cancer, the search for new, efficient and less toxic drugs remains a priority. In the 1950–1970s, a massive effort on isolation and screening of bacteria capable of producing bioactive secondary metabolites yielded a plethora of antibiotics, e.g. antibacterials vancomycin and erythromycin, as well as antifungal agents, e.g. nystatin and amphotericin B. Same can be said about the anticancer agents such as bacterially produced bleomycin and doxorubicin. However, these compounds, which have been developed into medically important drugs, mostly represent ‘low‐hanging fruits’ that were relatively easy to discover. Later on, efforts on finding new antibiotics faced a problem of consistent re‐discovery of already known compounds. The latter, together with high costs for screening, made pharmaceutical companies to invest into research and development of the drugs to treat chronic diseases, such as diabetes (Kuehn, 2011; Bartlett *et al*., 2013). Recent developments in the fields of genomics, bio‐ and chemoinformatics, metabolic engineering and synthetic biology opened completely new possibilities for drug discovery, thus re‐vitalizing interest in bacterial secondary metabolites. Combined with novel methods for isolation of rare, previously uncultivated bacteria, these approaches pave a new way towards better drugs. This review highlights recent developments in the bacterial bioprospecting and so‐called ‘genome mining’ (see below) and suggests how these can be interconnected in order to speed‐up and focus the drug discovery process. The schematic workflow showing integration of modern bacterial bioprospecting and genome mining is shown in Fig. 1.

## Bioprospecting of bacteria from natural environments

**Figure 2:**
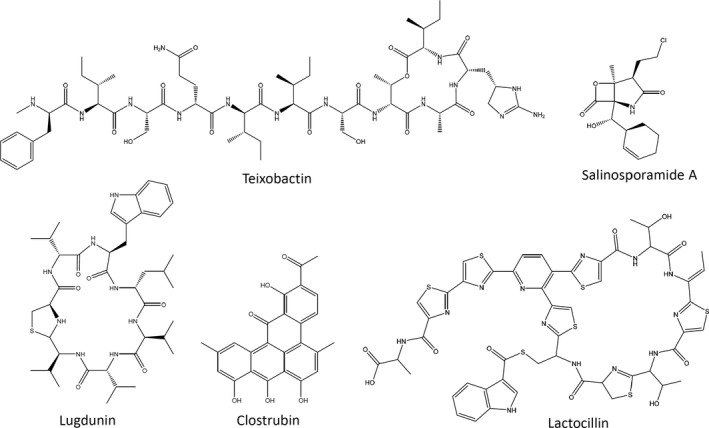
Novel bacterial secondary metabolites discovered via unconventional bioprospecting of previously uncharacterized bacteria isolated from unique environments.

At the onset of the antibiotic discovery era, which can be traced back to the 1940s as far as bacterial sources of antibiotics are concerned, much attention was given to actinomycete bacteria. These Gram‐positive bacteria represent one of the largest bacterial phyla and can be found in a wide variety of environments (van der Meij *et al*., 2017). The first antibiotic proved to be affective against the causative agent of tuberculosis (*Mycobacterium tuberculosis*), streptomycin, was discovered from a soil‐dwelling *Streptomyces griseus* (Smith and Waksman, 1947). A plethora of various bioactive compounds that were later developed into drugs were subsequently discovered from actinomycetes, including antifungal nystatin, antibacterials erythromycin, vancomycin, colistin (polymyxin E) and anticancer agent doxorubicin. The vast majority of these discoveries have been made in the 1950s–1970s, when pharmaceutical companies screened large numbers of actinomycetes mostly isolated from soil samples collected all over the world. However, the relatively easily isolated *Streptomyces* spp. dominated these collections, and repeated re‐discovery of already known compounds became a discouraging factor in further bioprospecting efforts. In order to increase the probability of finding new bioactive natural products, the search has turned to more exotic environments, such as marine sediments and animals, plants, insects and remote/extreme terrestrial locations (Hug *et al*., 2018). In addition, a considerable effort has been put into the development of methods for selective isolation of rare actinomycetes and cultivation of previously uncultivable bacteria (Puspita *et al*., 2012; Tiwari and Gupta, 2013). These efforts have indeed afforded many new bacterial genera and species, some of which were shown to produce novel bioactive compounds (Sherpa *et al*., 2015). Figure 2 shows examples of novel bacterial natural products discovered via bioprospecting of rare and unusual bacteria from various environments.

### Accessing rare and non‐obvious bacterial producers of secondary metabolites

Group of W. Fenical at the Scripps Institute (USA) was the first one to isolate a true marine actinomycete *Salinispora arenicola* (Maldonado *et al*., 2005), which was later found to produce salinosporamide A (Fig. 2), a novel secondary metabolite with proteasome‐inhibiting activity that is currently undergoing clinical trials in cancer patients under the name marizomib (Levin *et al*., 2016). Extensive metabolite profiling coupled to genome sequencing of this bacterium revealed other structurally unique natural products (Gontang *et al*., 2010), clearly supporting the idea that previously uncharted environments and novel bacterial species must be explored in search for new drug leads. At the same time, recent study by P. Jensen group from Scripps has clearly demonstrated that environment has a far greater impact on the ability of the same bacterial species to biosynthesize secondary metabolites (Ziemert *et al*., 2014). In this study, 75 isolates of the genus *Salinispiora* were obtained from geographically distinct areas, and subsequent genome sequencing and analyses revealed many gene clusters for the biosynthesis of secondary metabolites (BGCs) that were unique for isolates tagged to particular locations. This finding implies that actinomycetes are able to acquire new BGCs from other bacteria dwelling in the same environment, thereby probably enhancing their adaptive capabilities for a particular environmental niche. Other recent studies have also showed the same trend for *Strepomyces* spp., which were lately considered by many as being of no more interest in search for novel compounds. For example, the genome of *Streptomyces albus* isolated from a marine sponge was found to harbour many BGCs not found in its counterpart isolated from a terrestrial source (Ian *et al*., 2014). Although this marine isolate was not shown to actually produce novel secondary metabolites specified by these unique BGCs in the conditions tested, new approaches such as genome mining (see next sections) may activate silent BGCs and yield new bioactive compounds.

Although actinobacteria remain an interesting source for the drug discovery, research on these bacteria appears overcrowded and is undermined by frequent re‐discovery of already known compounds. This situation encouraged ‘out‐of‐the‐box’ approaches targeting other underexplored environmental niches and bacterial phyla. Recently, bioprospecting of human microbiota yielded two novel antibiotics, lugdunin and lactocillin (Fig. 2). Lugdunin, a thiazolidine‐containing cyclic peptide active against Gram‐positive bacteria, including pathogenic *Staphylococcus aureus*, was isolated from *Staphylococcus lugdunensis*, a common nasal commensal bacterium (Zipperer *et al*., 2016). Lactocillin is a novel thiopeptide antibiotic produced by the member of human vaginal microbiota *Lactobacillus gasseri* and active against vaginal pathogenic bacteria (Donia *et al*., 2014). Yet another example is represented by clostrubin and unusual antibiotic isolated from a strictly anaerobic bacterium *Clostridium beijerinckii* known for a long time as producer of organic solvents, such as butanol and acetone. By manipulating growth conditions, Hertweck group was able to induce formation of clostrubin (Fig. 2), a polyphenolic polyketide active against methicillin‐resistant *S. aureus*, vancomycin‐resistant *Enterococcus faecalis* and several *Mycobacterium* spp. (Pidot *et al*., 2014).

The use of novel approaches for culturing previously inaccessible bacteria is exemplified by the recent discovery of teixobactin (Fig. 2), a bactericidal antibiotic active against such pathogens as *S. aureus* and *M. tuberculosis*, reportedly without causing emergence of resistance (Ling *et al*., 2015). The producer of teixobactin, previously uncultivated bacterium provisionally named *Eleftheria terrae*, was discovered using a new tool, iChip, that allows to place individual bacterial cells from environmental samples into specially designed diffusion chambers, which are then deposited into the original environment to support proliferation. Other methods designed for selective isolation of particular bacterial phyla and genera rely mostly on a variety of media and special pretreatments, such as sample heating, microwave irradiation, bacteriophage and phenol treatment, are covered by several excellent reviews (Kurtböke, 2011; Tiwari and Gupta, 2013; Goodfellow *et al*., 2018).

Considering these examples, especially recent study on the actinomycetes from the Atacama Desert soils (Goodfellow *et al*., 2018), it is obvious that unique environments can provide bacterial species belonging to both well‐studied and underappreciated genera with unique biosynthetic capabilities. The latter can relatively easily be assessed via genomics and bio‐ and chemoinformatics, which have become indispensable for modern bacterial bioprospecting.

### Bacterial bioprospecting assisted by genomics and metagenomics

The examples given above comprise bacterial strains isolated using rather conventional methods and techniques. At the same time, metagenomics and single‐cell sequencing studies clearly demonstrate unprecedented potential of as yet uncultivated bacteria to produce secondary metabolites (Brady, 2007; Milshteyn *et al*., 2014; Bowers *et al*., 2017). Using this approach, the biosynthesis of several bioactive compounds previously discovered from marine animals was actually attributed to symbiotic bacteria associated with these hosts (Kwan *et al*., 2012; Schofield *et al*., 2015). Such examples include bryostatins, polyketides isolated from the marine bryozoan *Bugula neritina* with potential in anticancer therapy and treatment of Alzheimer disease, which are apparently produced by as yet uncultivated bacterial symbiont Candidatus *Endobugula sertula* (Davidson *et al*., 2001). Unfortunately, access to the biosynthetic potential of such bacterial symbionts outside of their natural habitats is rather limited. Although there are several examples of finding new secondary metabolites via cloning and expression of metagenomic DNA in various bacterial hosts (Gillespie *et al*., 2002; Parsley *et al*., 2011; Rebets *et al*., 2017), as well as upon heterologous expression of BGCs (see next section), this approach has many hurdles. The latter can be exemplified by poor quality of metagenomics DNA, necessity to screen very large metagenomics libraries, potential inability of the host's transcriptional machinery to recognize regulatory sequences in foreign DNA, the absence of particular biosynthetic precursors, etc. Thus, new cultivation techniques that would enable isolation and maintenance of previously uncultivated bacteria may greatly assist in gaining access to novel bacterial species and their secondary metabolites.

The efforts on bacterial bioprospecting can be greatly assisted by modern DNA sequencing technologies (later called NGS – next‐generation sequencing) that offer new opportunities for discovering habitats of rare bacteria that may become sources of new drug leads. One of the NGS‐based approaches targeted at environmental samples encompasses metagenome sequencing, allowing direct access to the genomes of the majority of bacteria dwelling in this habitat. Whether or not these bacteria can be cultivated is of no importance, as long as the genes for secondary metabolite biosynthesis discovered in metagenomes can be amplified or synthesized and heterologously expressed in an appropriate well‐characterized bacterial host (see next section). However, considering relatively low success rate for heterologous expression of metagenomic DNA, it is still preferable to be able to isolate and cultivate an actual host harbouring secondary metabolite biosynthesis genes and thus most likely possessing all the machinery required for the biosynthesis of cognate compounds. In this, metagenomics may also be of great help, since it can provide important insights into the metabolic/nutritional requirements of particular bacteria, especially if their genomes can be at least partially assembled from the metagenomics data. If such bacterium carries biosynthetic genes that are presumed to specify biosynthesis of a new secondary metabolite, such information can be used to design growth media and specific conditions allowing isolation and propagation of this bacterium.

All the bioprospecting efforts aimed at isolation and cultivation of prospective bacterial producers of bioactive secondary metabolites face the challenge of de‐replication. The latter aims at elimination of isolates and compounds that are already known and focusing only on those that are potentially novel. Historically, 16S rRNA gene sequence‐based taxonomy was used to de‐replicate bacterial isolates (Brandao *et al*., 2002), and for a long time, it was assumed that bacteria with > 99% sequence identity represent the same species and are likely to produce the same secondary metabolites. However, studies based on genomics revealed that this assumption is not entirely correct and may lead to elimination of valuable isolates. For example, comparative genome analysis of the marine sponge‐derived *Actinoalloteichus fjordicus* that shares 99.9% 16S rRNA gene sequence identity with that of *Actinoalloteichus hyamenocidonis* isolated from a different sponge showed drastically different capacities of these bacteria to produce secondary metabolites (Nouioui *et al*., 2017).

Hence, more effort must be directed towards chemical de‐replication, when bacterial extracts are screened using state‐of‐the‐art analytical methods allowing identification of known compounds in these complex mixtures. This would allow, for example, efficient identification of unique compounds in bacterial species that share very similar 16S RNA gene sequences but originate from different environments and thus might possess different biosynthetic capabilities. Since recent comprehensive reviews on the issues of de‐replication describing novel methods such as hybrid NMR/high‐resolution mass spectrometry (Gomes *et al*., 2018) and comparative metabolomics (Covington *et al*., 2017) are available, these topics will not be covered in this mini‐review.

## Mining of bacterial genomes for new bioactive natural products

The fast development of genome sequencing technologies and power of advanced computational analyses of the DNA sequences dramatically increased the potential for natural product discovery. Many bioactive bacterial secondary metabolites are biosynthesized by multi‐modular enzymes, such as polyketide synthases and non‐ribosomal peptide synthetases (NRPS), to name the most common types. The genes encoding biosynthetic machinery for secondary metabolites are organized in biosynthetic gene clusters (BGC), analyses of which can provide important information regarding chemical class of the compounds they specify. This predictive correlation between BGCs and cognate compounds can now be achieved using a wide range of bioinformatics tools [e.g. antiSMASH (Weber *et al*., 2015), BAGEL3 (van Heel *et al*., 2013) and PRISM (Skinnider *et al*., 2016)]. It became then increasingly clear, that some bacteria are able to produce far greater numbers of secondary metabolites than a conventional screening can reveal. Indeed, genomes of actinomycete bacteria may contain up to 50 different BGCs, many of which are uncharacterized, and may yield novel bioactive compounds. The majority of such BGCs appear to be ‘silent’ in the laboratory conditions or their cognate metabolites are produced in very small amounts, not sufficient for proper characterization or industrial applications.

**Figure 3:**
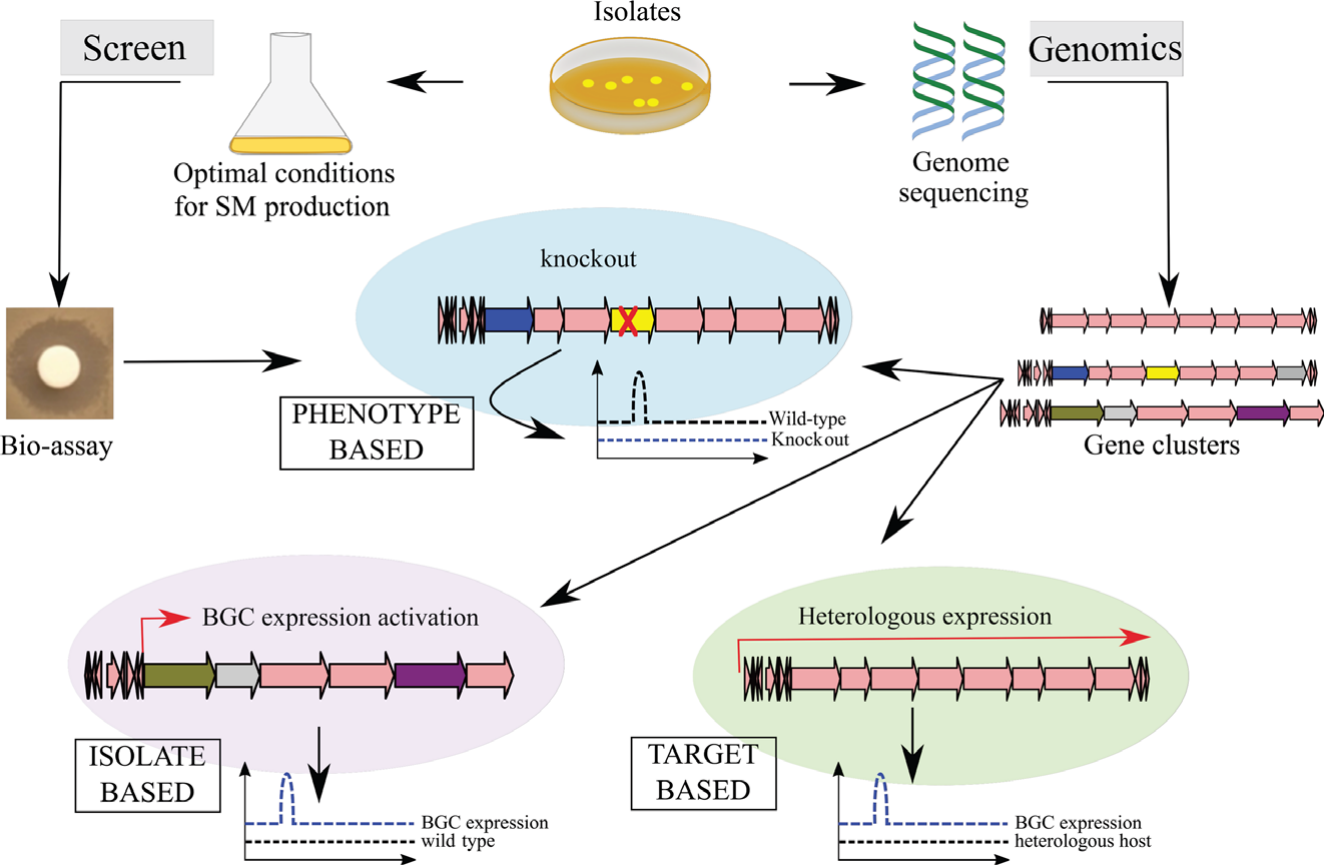
Different approaches used in mining bacterial genomes for novel natural products (see text for details).

**Figure 4:**
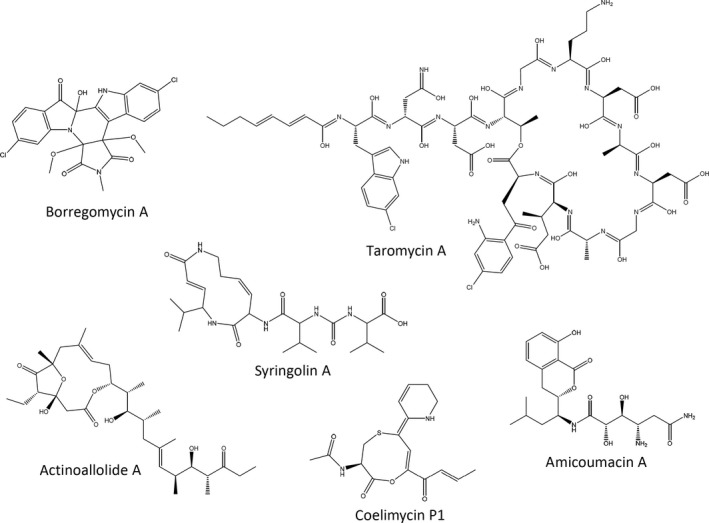
Examples of novel bioactive bacterial secondary metabolites discovered using various approaches of genome mining (see Table 1 for details).

Our knowledge on the biosynthesis of secondary metabolites by bacteria is constantly expanding, thereby adding new information that connects biosynthetic genes with metabolites of particular chemical classes. In this respect, recently developed standardized database of BGCs, MIBiG, represents an important resource allowing identification of both already characterized BGCs and those that may encode enzymes for assembly of novel secondary metabolites (Epstein *et al*., 2018). Supported by the abovementioned bioinformatics tools and databases, a new concept of so‐called ‘genome mining’ based on analysing bacterial genomes for the presence of various BGCs has been established. Figure 3 shows how genome mining approach can be applied in different ways based upon (i) phenotype of the producer (e.g. bioactivity and biosynthesis of specific compounds or enzymes); (ii) targeted chemical class of natural products (e.g. terpenes, lasso peptides and aromatic polyketides) and (iii) the potential uniqueness of the bacterial isolate that may yield novel compounds (e.g. novel or taxonomically divergent species, and isolates from unique environmental sources). A starting point for application of target‐based genome mining is the choice of a specific chemical class of a natural product, followed by mining of genomic DNA sequence for genes encoding enzymes that specify biosynthesis of corresponding molecular scaffold. Once the candidate target BGCs are detected, different techniques can be used to identify the secondary metabolite it specifies and to increase its production to a level allowing purification and biological testing. The expression of this BGC could be activated or enhanced in the host organism by genetic manipulation and/or by optimization of the growth conditions. Alternatively, this BCG can be cloned and heterologously expressed in a well‐characterized or engineered host. The goals of the genome mining based on phenotype and uniqueness of the bacterial isolate are self‐explanatory and do not require further specification. Instead, in the following subsections, we would like to focus on different strategies that can be employed to reach particular goals of the genome mining. Examples of novel bioactive secondary metabolites discovered via various types of genome mining are shown in Fig. 4 (see Table 1 for details).

**Table 1:** Selected examples of novel bacterial secondary metabolites discovered via genome mining approaches

Compound	Class	Activity	BGC original host	Identification strategy	Reference
Scleric acid	(2‐(benzoyloxy) acetyl)‐L‐proline	*Mycobacterium tuberculosis*	*Streptomyces sclerotialus* NRRL ISP‐5269	Heterologous expression in *Streptomyces albus* J1074	(Alberti *et al*., 2019)
Enterocin	Polyketide	Cytotoxic	*Salinispora pacifica* CNT‐150	Heterologous expression in *Streptomyces coelicolor* M1146	(Bonet *et al*., 2014)
Borregomycin A	Indolotryptoline	CaMKIIδ kinase inhibition	Metagenomic DNA from Anza‐Borrego Desert soil	Heterologous expression in *Streptomyces albus* J1074	(Chang and Brady, 2013)
Avermectins	Polyketides	Antihelminthic	*Streptomyces avermitilis* ATCC 31267	Heterologous expression in *Streptomyces lividans* 1326	(Deng *et al*., 2017)
Tetarimycin A	Polyketide	MRSA	Metagenomic DNA	Heterologous expression in *Streptomyces albus* J1074	(Kallifidas *et al*., 2012)
Cosmomycins	Polyketides	Cytotoxic	*Streptomyces* sp. CNT‐302	Heterologous expression in *Streptomyces coelicolor* M512	(Larson *et al*., 2017)
Alterochromide	Lipopeptide	Antibacterial, cytotoxic	*Pseudoalteromonas piscicida* JCM 20779	Heterologous expression in *Escherichia coli* BL21(DE3)	(Ross *et al*., 2015)
Taromycin A	NP peptide	Antibacterial	*Saccharomonospora* sp. CNQ490	Heterologous expression in *Streptomyces coelicolor* M512	(Yamanaka *et al*., 2014)
Streptoseomycin	Polyketide	*Helicobacter pylori*	*Streptomyces seoulensis* A01	Heterologous expression in *Streptomyces chartreusis* 1018	(Zhang *et al*., 2018)
Actinoallolides	Polyketides	Anti‐trypanosomal	*Actinoallomurus fulvus* MK10‐037	Heterologous expression in *Streptomyces coelicolor* M1152	(Inahashi *et al*., 2018)
Thaxtomins	Nitrated diketopiperazines	Herbicide	*Streptomyces scabiei* 87.22	Heterologous expression in *Streptomyces albus* J1074	(Jiang *et al*., 2018)
Syringolin	NR peptide	Cytotoxic	*Pseudomonas syringae pv. syringae* (Pss) B728a	Heterologous expression in *Streptomyces lividans* TK24	(Huang *et al*., 2018)
Pseudomycoicidin	Lantibiotic	Antibacterial	*Bacillus pseudomycoides* DSM 12442	Heterologous expression in *Escherichia coli* BL21(DE3)	(Basi‐Chipalu *et al*., 2015)
Amicoumacin	Dihydroisocoumarin	Antibacterial	*Bacillus subtilis* 1779	Heterologous expression in *Bacillus subtilis* JH642	(Li *et al*., 2015)
Pyxidicyclines	Polyketides	Topoisomerase inhibitor	*Pyxidicoccus fallax* An d48	BGC activation and heterologous expression	(Panter *et al*., 2018)
Chattamycins	Polyketides	Cytotoxic	*Streptomyces chattanoogensis* L10	Overexpression of pathway‐specific activator	(Zhou *et al*., 2015)
Stambomycins	Polyketides	Cytotoxic	*Streptomyces ambofaciens* ATCC 23877	Overexpression of pathway‐specific activator	(Laureti *et al*., 2011)
Coelimycin P1	Polyketide	Antibacterial	*Streptomyces coelicolor* A3(2)	Inactivation of pathway‐specific repressor	Gomez‐Escribano *et al*., 2012;
Gacamide A	Lipopeptide	Antibacterial	*Pseudomonas fluorescens* Pf0‐1	Repair of defective pathway‐specific activator	Jahanshah *et al*., 2019;
Atolypenes	Sesterterpenes	Cytotoxic	*Amycolatopsis tolypomycina* NRRL B‐24205	Cas9‐TAR BGC refactoring	Kim *et al*., 2018

### Host engineering and heterologous expression of BGCs

Although the idea of heterologous production of a secondary metabolite is not new, only recently a significant progress has been made in this approach, mainly due to the use of engineered bacterial hosts. Quite often, bacterial producers of new bioactive compounds are barely cultivable and cannot be used in the industrial setting that requires good growth characteristics and yields allowing profitable operation. The robust, fast‐growing bacterial host able to biosynthesize a wide spectrum of compounds can be used for heterologous expression of entire biosynthetic pathways from such ‘problematic’ bacteria. *Escherichia coli* and the representatives of genus *Streptomyces* are known as suitable heterologous hosts for this purpose. Although *E. coli* is easy to cultivate and a well‐developed toolbox for gene manipulations is available for this bacterium, many secondary metabolites cannot be easily produced in this host because of the lack of specific precursors, sigma factors for the recognition of alien promoters, problems with protein folding or toxicity of the final products. From this point of view, *Streptomyces* bacteria have the advantages of availability of pathways for the biosynthesis of many precursors, post‐translational modification machinery and genes for resistance against a variety of toxic compounds such as antibiotics. However, streptomycetes often produce their own secondary metabolites, thus channelling away precursors needed for the biosynthesis of an exogenous compound, leading to the decreased yield and complicating the purification. Thus, elimination of the competing endogenous pathways by deleting corresponding BGCs became the major theme in construction of several ‘bacterial chasses’ for heterologous expression.

Such hosts based on *Streptomyces coelicolor* M145 have been constructed (Gomez‐Escribano and Bibb, 2011) by deleting four antibiotic BGCs (for actinorhodin, undecylprodigiosin, calcium‐dependent antibiotic and coelimycin) to increase the precursors pool and simplify the metabolic profile. In addition, point mutations into the genes *rpoB* and *rpsL* were introduced to pleiotropically increase the production of secondary metabolites via so‐called ribosome engineering (Ochi *et al*., 2004). To assess the efficiency of new surrogate host, exogenous BGCs for chloramphenicol from *Streptomyces venezuelae* ATCC 10712 and congocidine from *Streptomyces ambofaciens* have been introduced into the engineered strain. Further analysis confirmed not only the heterologous expression of both clusters, but also the elevated, in comparison with the native strains, level of production of chloramphenicol and congocidine. Two of these engineered *S. coelicolor* hosts, M1152 and M1154, have later been also found useful for heterologous expression of a wide spectrum of BGCs, such as the ones for indolocarbazole, aminocoumarines, liponucleoside and thiopeptides (Flinspach *et al*., 2010, 2014; Li *et al*., 2013; Linares‐Otoya *et al*., 2017). Additional engineering steps have been performed in order to make *S. coelicolor* M1152 to efficiently produce compounds biosynthesized by type III PKS (Thanapipatsiri *et al*., 2015). For this purpose, three endogenous type III PKS genes have been removed from the chromosome to make a clean background for heterologous expression of type III PKS genes from different organisms. Such mutant strain, designated M1317, was validated by the production of germicidin and flaviolin after reintroduction of germicidin synthase gene *gcs* of *S. coelicolor* and *rppA* homologue from *S. venezuelae* respectively.

*Streptomyces lividans* is closely related to *S. coelicolor* and is successfully used as a host for heterologous production of both proteins (Anné and Van Mellaert, 1993) and secondary metabolites (Eustaquio *et al*., 2005). Recently, a set of *S. lividans* TK24 mutants, designated RedStrep strains, have been developed by sequential deletions of clusters predicted to be potentially interfering with the production of antitumoral polyketide mithramycin A (MTM) (Nováková *et al*., 2018). The entire biosynthetic gene cluster of mithramycin A from *Streptomyces argillaceus* ATCC12956 was introduced into *S. lividans* TK24 wild type and RedStrep strains. While parent strain TK24 was able to produce MTM in amount of 850 mg l^−1^, the deletion of actinorhodine, undecylprodigiosin and calcium‐dependent antibiotic BGCs resulted in the production of almost 3 g l^−1^ MTM, which by far surpassed the production of MTM in the native strain *S*. *argillaceus*.

Significant genome reduction in *Streptomyces avermitilis* has been achieved via a series of sequential large deletions, yielding a new heterologous production host (Komatsu *et al*., 2010). The region of ca. 1.4 Mb containing the BGCs for major metabolites produced by the parent strain was deleted. To confirm the suitability of the new hosts for heterologous production, exogenous BGCs for streptomycin from *S. griseus* IFO 13350*,* cephamycin C from *Streptomyces clavuligerus* ATCC 27064 and pladienolide from *Streptomyces platensis* 11107 have been introduced into the wild type and engineered strains. The first two metabolites were produced in mutants at levels higher than in the wild‐type *S. avermitilis* and also by the native producers. Pladienolide at first was not produced, but the problem was alleviated by introduction of a copy of the regulatory gene *pldR* under control of an alternative promotor. Engineered *S. avermitilis* strains SUKA17 and SUKA22 were later used to express more than 20 BGCs for secondary metabolites with different chemical structures and from different hosts (Komatsu *et al*., 2013).

Recently, a new host based on genome‐reduced *S. albus* J1074 has been reported (Myronovskyi *et al*., 2018). Deletion of 15 BGCs from the chromosome of *S. albus* yielded strain Del14, which utility for heterologous expression was confirmed for BGCs specifying chemically diverse metabolites (PKS types I and II, glycosylated phospholipid, lantibiotic, nucleoside). The strain Del14 showed enhanced production of secondary metabolites compared to *S. albus* J1074 and *S. coelicolor* M1152 and M1154. Additional changes in the genome of Del14 have been made by introduction of phage *phi*C31 *attB* attachment sites, yielding strains B2P1 and B4. This step allowed the integration of multiple copies of heterologous BGCs into the chromosome and resulted in improved yields of six out of eight exogenous compounds tested.

In parallel with the engineering of heterologous hosts, novel methods for cloning of entire BGCs were rapidly developing over the last decade. Previously, most of the BGCs were assembled using lambda‐red ‘recombineering’ (Gust *et al*., 2004), a robust but labour‐ and time‐consuming procedure. Transformation‐associated recombination in yeast (Kim *et al*., 2010) offered an excellent alternative for the assembly of large BGCs either from several overlapping PCR products encompassing entire BGC (Bilyk *et al*., 2016), by direct capture of the whole BGC (Yamanaka *et al*., 2014), or assembly from restriction fragments isolated from a genomic library covering entire BGC (Nováková *et al*., 2018). A prerequisite for the successful cloning/assembly of large BGCs in yeast was construction of specialized ‘capture’ vectors capable of replicating in *E. coli* and yeast, and integrating into the genome of the host chosen for heterologous expression. Details on various modern methods currently employed for this purpose can be found in recent reviews (e.g. Luo *et al*., 2016).

Several other examples on successful heterologous expression of the BGCs, including ones accomplished in hosts other than streptomycetes, are presented in Table 1. Those include targeted genome mining leading to the discovery of pseudomycoicidin from *Bacillus pseudomycoides* DSM 12442 via heterologous expression of its BGC in *E. coli* (Basi‐Chipalu *et al*., 2015). Here, the NCBI database was specifically searched for proteins with high homology to mersacidin biosynthetic enzyme (MrsM), which is involved in post‐translational modification of the pre‐peptide (Altena *et al*., 2000). *In silico* analysis revealed a new class II lantibiotic gene cluster in *B. pseudomycoides* DSM 12442. Putative new BGC for this lantibiotic was heterologously expressed in *E. coli* BL21, and its production was detected via matrix‐assisted laser desorption ionization–time‐of‐flight mass spectrometry (MALDI‐TOF MS) (Basi‐Chipalu *et al*., 2015). Interestingly, 6 years before discovery of pseudomycoicidin, the same group followed this procedure for detection of potentially new lantibiotics in NCBI database and was able to identify novel two‐peptide lantibiotic lichenicidin in *Bacillus licheniformis* DSM 13. However, in this work, new peptides were produced in *B. licheniformis* host and the compound‐BGC connection was determined using gene knockout in the original host (Dischinger *et al*., 2009).

Applying isolate‐based genome mining approach (see Fig. 3), genomes of bacteria obtained from unusual environments can be scanned for unique BGCs that may govern biosynthesis of novel natural products. A good example of such isolate‐based genome mining is the identification of a lipopeptide‐type antibiotic taromycin A from *Saccharomonospora* sp. CNQ‐490 isolated from marine sediments collected at the mouth of the La Jolla Submarine Canyon. Bioinformatics analysis of the 4.9‐Mb draft genome sequence of this bacterium revealed 19 BGCs, indicating a potential to produce diverse secondary metabolites (Yamanaka *et al*., 2014). BGC for taromycin A is silent in *Saccharomonospora* sp. CNQ‐490, and its expression was carried out in heterologous host *S. coelicolor* M512. In this case, a forced expression of the pathway‐specific regulator was necessary to achieve production of taromycin A, which demonstrated moderate calcium‐dependent activity against several Gram‐positive bacteria (Yamanaka *et al*., 2014).

Whether the BGC of interest is present in the native host, or transferred to a heterologous one, its expression can be activated by various methods. It can be achieved via supplementing growth media with elicitors (Xu *et al*., 2018), by targeting global regulators (Huang *et al*., 2016) or pathway‐specific regulators (Tu *et al*., 2018). Some of these strategies are discussed in more details below.

### Genes‐to‐compound connection

When, upon genome analysis, a potentially novel BGC is discovered, its product may be identified upon inactivation of a particular biosynthetic gene coupled with comparative metabolic profiling of the wild type and mutant strains using HPLC and/or LC‐MS. This strategy was used for the discovery of coelichelin from *S. coelicolor*, when a cryptic NRPS gene cluster was inactivated and the association of this BGC to its product could be made using comparative metabolomics (Lautru *et al*., 2005). This approach does not require the compound specified by a cryptic BGC to be bioactive and relies solely on the comparison of metabolic profiles. This can be advantageous, since bioactivity of a new compound may not be readily detectable. Once a novelty is established, the new compound can be purified and sent to many partners that may have complementary assays at hand. At the same time, genome mining combined with a phenotype brought about by the production of a particular metabolite is rather straight forward and allows to connect BGC with the compound of interest. For example, bioassays can be combined with a phenotype‐based genome mining approach (Fig. 3) when a particular bacterial strain produces as yet uncharacterized bioactive compound for which neither chemical structure nor biosynthetic pathway is known.

Recently, *Streptomyces* sp. NP10 strain was isolated, which is characterized by unusually high production of free fatty acids (FAs) (Ilic‐Tomic *et al*., 2015). A lipidomics study on this strain revealed a large structural diversity of produced FAs with over 50 different *n* and branched chain, (un)saturated and cyclopropane FAs (C_7_–C_30_). Two of these, *i*‐17:0cy9‐10 and *a*‐18:0cy9‐10, represent new natural products (Ilic‐Tomic *et al*., 2015). Based on the genome analysis of this strain coupled to gene inactivation, our group successfully identified a unique FA biosynthesis gene cluster responsible for this particular phenotype (Schneider *et al*., 2018a).

Using the same approach, we were also able to make a clear connection between antibacterial activity of *Streptomyces* sp. YIM 130001 isolated from lichen, the compound responsible for this activity and a cognate BGC (Schneider *et al*., 2018b). The identified bioactive compound turned out to be a thiopeptide closely related to geninthiocin A, for which BGC has not yet been described. Analyses of the genes in the identified BGCs showed them to be very similar to those involved in the biosynthesis of another closely related thiopeptide antibiotic, berninamycin (Malcolmson *et al*., 2013).

Genome mining aimed at connecting compounds to particular BGCs was also recently used for myxobacterium *Chondromyces crocatus* Cm c5, which produces judazoles, chondramides, crocacins, chondrochlorens, thuggacins, crocapeptins and crocagins. All the secondary metabolites mentioned above were presumed to be synthesized by NRPS. Analysis of the genome sequence revealed the presence of at least 11 NRPS BGCs, most of which must be cryptic, and it was not possible to connect them to the cognate compounds based on bioinformatics only. All 11 BGCs were inactivated by gene disruptions, and resulting recombinant strains subjected to the secondary metabolomics study, which allowed identification of BGC for crocadepsins (Surup *et al*., 2018).

### Manipulation of regulatory genes and promoters

The vast majority of BGCs for secondary metabolites contain pathway‐specific regulatory genes, which can be represented by transcriptional activators and/or repressors. It is logical to assume that some BGCs may be not expressed due to the lack of expression of activators or constitutive expression of repressors. In a pioneering study by Gottelt *et al*. (2010), researchers deleted a gene encoding ScbR, a γ‐butyrolactone receptor‐like protein within a cryptic type I PKS gene cluster *cpk* in a model streptomycete bacterium, *S. coelicolor* A3(2). Apparently, this protein acted as a repressor, since its deletion led to *cpk* cluster activation and production of an antibacterial compound later identified as coelimycin P1 (Gomez‐Escribano *et al*., 2012).

Laureti *et al*. (2011) demonstrated how expression of a pathway‐specific regulator can lead to activation of a silent BGC and result in a discovery of novel bioactive natural product. In this study, genome sequence of *S. ambofaciens* ATCC 23877 was analysed, revealing, among other BGCs, an unusually large type I PKS cluster, which harboured a gene for LuxR‐type regulator. Expression of this regulator from a constitutive promoter apparently activated this gene cluster, and led to the discovery of a series of 51‐membered glycosylated macrolides, stambomycins, which exhibited anti‐proliferative activity.

This strategy for activation of silent BGCs was further developed by several research groups, which also used promoter replacement as an alternative approach for the clusters that do not contain any obvious pathway‐specific regulatory genes. Luo *et al*. (2013) identified a silent PKS‐NRPS gene cluster in the genome of *S. griseus* and used a combination of PCR and DNA synthesis to assemble it in yeast by means of transformation‐associated recombination. Importantly, all the biosynthetic genes in this semi‐synthetic cluster were placed under control of a constitutive promoter ermE*p. The refactored cluster was then introduced into *S. lividans*, and recombinant strain was shown to produce new polycyclic tetramate macrolactams (Luo *et al*., 2013).

Lately, a combination of several methods of genome mining is gaining momentum, exemplified by the example of *S. albus* J1074, a well‐known host frequently used for heterologous expression of exogenous BGCs. Olano *et al*. (2014) have selected five BGCs in the genome of this bacterium, which they manipulated by either inserting strong constitutive promoter upstream of the biosynthetic genes, overexpressing pathway‐specific regulators or gene deletion. This strategy has led to isolation of novel polycyclic tetramate macrolactams, activation of candicidin and antimycin biosynthesis, and identification of paulomycins BGC (Olano *et al*., 2014). The same group further expanded this approach to genome mining of *Streptomyces agrillaceus* by repressor inactivation and engineering precursor supply, which in combination led to the discovery of six new alkaloids (Ye *et al*., 2017).

## Engineering of bacterial natural product biosynthesis pathways

Bacterial secondary metabolites have evolved to serve a particular biological function that is beneficial for the producer, while not necessarily being optimal for use as human drugs. Poor solubility and pharmacokinetics, general toxicity and off‐target effects may prevent bioactive bacterial natural products from being developed into drugs. Although modifications by means of synthetic chemistry or total synthesis to generate analogues with improved drug‐like properties are viable strategies to overcome these hurdles, such approaches are often cost‐prohibiting, too complicated or even impossible for particular molecular moieties. In the past three decades, an alternative approach of biosynthetic engineering has emerged that is based on solid knowledge of the biosynthetic pathway leading to a particular metabolite and the ability to manipulate it in a way that leads to production of a desired analogue. One of the first examples of such engineered compounds was analogues of macrolide antibiotics, which are synthesized by multi‐modular type I PKS. The biosynthetic logic of these enzymes [for review, see (Weissman, 2004)] allows to make predictable changes in the macrolactam rings of these antibiotics, thereby generating novel compounds with altered ring size and degrees of reduction (Weissman, 2016). Although the biosynthetic engineering principle was first tested on erythromycin, an antibacterial macrolide antibiotic from *Saccharopolyspora erythraea* (Donadio *et al*., 1993), no clinically useful analogues with improved activity were reported. The precedent, however, prompted others to use biosynthetic engineering in order to improve pharmacological properties of bacterial natural products. One of such examples is provided by the biosynthetic engineering of nystatin, an antifungal polyene macrolide produced by *Streptomyces noursei* ATCC 11455, and used since 1950s to treat superficial fungal infections. The main disadvantage of nystatin is its toxicity upon systemic administration, which could lead to severe side‐effects. The nystatin BGC encompassing six modular PKS type I and accessory genes was cloned and genes’ functions determined by gene knockouts and bioinformatics analyses (Brautaset *et al*., 2000). When the biosynthetic machinery behind nystatin biosynthesis was understood, specific parts of its molecule, in particular, polyene region, carboxyl group and the polyol region, have been changed by genetic engineering of corresponding enzymes. A series of genetically engineered nystatin analogues have been generated and tested *in vitro* for antifungal and haemolytic activities (Brautaset *et al*., 2008). The data acquired on structure–activity–toxicity allowed the selection of the most potent and less toxic compounds. *In vivo* tests further revealed that two of the analogues, BSG005 and BSG020, performed better than amphotericin B, the only polyene macrolide antibiotic currently used to treat systemic fungal infections. The BSG005 analogue is currently in late preclinical development as an antifungal drug for human therapy.

Another example, also taking advantage of the modular enzyme architecture, is represented by daptomycin, the lipopeptide antibiotic produced by *Streptomyces roseosporus* currently used (under trade name Cubicin) to treat systemic infections caused by Gram‐positive bacteria. Daptomycin is synthesized by the modular NRPS, making its biosynthetic pathway amenable to genetic modifications by swapping the domains, modules and subunits in an approach called ‘combinatorial biosynthesis’ [for review, see (Kries, 2016)]. Multiple attempts to engineer daptomycin NRPS have been made by combining gene deletions and multiple module exchanges, generating a library of ca 120 new lipopeptides, over 40 of which have been produced in quantities allowing further investigations on their structures and antibacterial activities. Some of them indeed demonstrated better pharmacological properties by having the activity similar to daptomycin but lower mammalian toxicity (Doekel *et al*., 2008; Nguyen *et al*., 2010).

Combinatorial biosynthesis is not restricted to modular enzymes’ engineering. This approach has also been applied to mithramycin, glycosylated aromatic polyketide used for treatment of several types of cancer, but known to have a severe toxicity. The mithramycin BGC has been cloned from *S. argillaceus* ATCC 12956, and its biosynthetic pathway was characterized. The information on the pathway was used to genetically engineer the cluster by changing mithramycin glycosylation profile via sugar moiety modifications or substitutions and combinations of different approaches, including bioconversions and lipase‐based biocatalysis (Remsing *et al*., 2003; Baig *et al*., 2008). These efforts led to the generation of more than 90 mithramycin analogues, among them compounds with lower toxicity and higher activity, which were chosen as candidates for drug development. The clinical trial for improved mithramycin analogues is currently under way (Núñez *et al*., 2012; Méndez *et al*., 2015).

## Application of synthetic biology tools for drug discovery in bacteria

Synthetic biology, a new discipline which emerged quite recently (Keasling, 2012), involves application of engineering principles to biological systems in order to build new biological systems and organisms with pre‐programmed features. Synthetic biology focuses on the modelling and construction of standard interchangeable parts and devices (promoters, Shine–Dalgarno sequences, ORFs, terminators, genetic circuits) and assembling them into new biological systems such as novel metabolic pathways. Using the principles and technologies of synthetic biology, bacterial BGCs can be redesigned, the new pathways introduced into the hosts pre‐engineered for biosynthesis of particular compounds specified by this BGC, and used in a plug‐and‐play manner (Medema *et al*., 2011).

Reorganizations of metabolic pathways, also termed ‘refactoring’, must take into account the levels of gene expression. Ideally, enzymes should be produced only in the amounts sufficient to catalyse specific reactions, and excessive synthesis of enzymes is too energy‐demanding or could be even toxic for the cell. Gene expression in bacterial BGCs is usually tightly regulated by a complex system consisting of several layers of regulation (Liu *et al*., 2013). The regulatory synthetic devices or synthetic genetic circuits control the target biological process by sensing the environmental signals and responding accordingly. Nowadays, many synthetic genetic circuits offer new possibilities for controlling BGCs, including those based on ribozymes (Shen *et al*., 2015), riboswitches (Serganov and Nudler, 2013) and inducible dual control systems, where the interchangeable inducible promoters of different strength are induced by a range of inducer concentrations, allowing tuning of gene expression in bacteria (Brophy and Voigt, 2014; Horbal and Luzhetskyy, 2016). Although application of these circuits for controlling secondary metabolism in bacteria is currently still in the early stage, they will certainly become valuable in the future biotech industry, especially for controlled production of valuable drugs.

Synthetic biology also offers possibilities for construction of *in vivo* biosensors, the synthetic devices for selection of strains producing specific compounds and intermediates as a tool that greatly simplifies genome mining and screening. Biosensors reacting to production of specific secondary metabolites can replace laborious and time‐consuming analytical chemistry needed to analyse the results of genome mining. Certain types of biosensors are based on proteins which are able to translate the chemical information (target compound) into fluorescent or colorimetric signal, which makes the screening fast and convenient (DeLoache *et al*., 2015). Recently, several attempts to build biosensors reacting to target compounds and allowing selection of high‐producing strains have been reported. Based on the TetR‐like repressors, which are very abundant in the BGCs, and *bpsA* reporter gene encoding indigoidine synthetase, the proof of principle was obtained by detecting production of target antibiotic coelimycin by a biosensor (Sun *et al*., 2017). In another case, the constructed biosensor was used to improve production of antibiotic pamamycin (Reberts *et al*., 2018). In this work, the biosensor built based on the native TetR‐like repressor proved to be suboptimal, but was then fine‐tuned by altering the promoter and operator regions of the reporter, as well as the ligand affinity of the repressor. Although the attempts were successful, and much useful information has been obtained, these tools still need to be improved to make them robust and applicable to a wide variety of BGCs that are still not characterized with respect to their products.

Synthetic biology also inspired application of the CRISPR/Cas9 genome editing technologies for the discovery of new natural products in bacteria. (Cobb *et al*. (2014) applied this technique for *Streptomyces* gene editing, deleting the entire undecylprodigiosin BGC from the chromosome of *S. lividans* 66. A year later, several groups demonstrated the efficacy of the method by performing single and multiple gene deletions and point mutations in the genomes of *Streptomyces* spp. (Huang *et al*., 2015; Zeng *et al*., 2015). There is also a progress in application of the system for CRISPR/Cas9‐mediated activation of cryptic BGCs in *Streptomyces* (Zhang *et al*., 2017), manipulating the BSGs (Liu *et al*., 2015) and cloning and refactoring of BGCs for heterologous expression. Cloning the big BGCs of 100 kb or larger is still a bottleneck in heterologous expression. In recent studies, CRISPR/Cas9 approach was combined with Gibson assembly and TAR cloning allowing to clone up to 100 kb of intact DNA, improve the efficiency of TAR and even assemble megabase‐sized fragment of DNA by homologous recombination assembly (CasHRA) (Jiang *et al*., 2015; Wang *et al*., 2015, 2016; Zhou *et al*., 2016). Considering the above, CRISPR/Cas9‐based methodologies appear to be very promising tools for the discovery of novel bioactive secondary metabolites in bacteria.

## Conclusion

Despite a clear scepticism from the pharmaceutical companies regarding bacterial natural products, most of which traditionally been antibiotics, a considerable effort is being made by scientific community to prove that many new drugs can still be developed from bacterial secondary metabolites. New ways of how previously uncultivable bacteria can be isolated, exploration of unique environments, and metagenomic‐assisted isolation methods will certainly provide bacterial producers of new bioactive compounds. Recent developments in analytic techniques allowing fast de‐replication and identification of compounds’ chemical classes and structural features will greatly support the bacterial bioprospecting for new drugs. Development and application of new assays for bioactivities (e.g. for quorum sensing inhibitors, pain suppressors and bioactive compounds against Alzheimer disease) will help to identify compounds that were previously not detected simply because there were no corresponding assays. Genome mining will definitely expand to more bacterial species and BGCs, providing new bioactive compounds via a combination of different methods, including those inspired by synthetic biology. Once discovered, the new compounds will have to be produced at a scale allowing biological testing for pharmacological properties. Therefore, the supply issue will have to be addressed by both refactoring of the BGCs and streamlining metabolism of the host through metabolic engineering, focusing on metabolic flux through target pathway, robustness of the producer and balancing precursor supply. Considering all the advances made in bacterial biology over the last two decades, the future for the discovery of novel drug candidates from bacteria looks bright.

## Conflict of interest

Authors declare that they do not have conflict of interest.
